# Predictability Bounds of Electronic Health Records

**DOI:** 10.1038/srep11865

**Published:** 2015-07-07

**Authors:** Dominik Dahlem, Diego Maniloff, Carlo Ratti

**Affiliations:** 1 IBM Research–Ireland, Dublin 15, Ireland; 2 Senseable City Lab, Massachusetts Institute of Technology, Cambridge, MA 02139, USA

## Abstract

The ability to intervene in disease progression given a person’s disease history has the potential to solve one of society’s most pressing issues: advancing health care delivery and reducing its cost. Controlling disease progression is inherently associated with the ability to predict possible future diseases given a patient’s medical history. We invoke an information-theoretic methodology to quantify the level of predictability inherent in disease histories of a large electronic health records dataset with over half a million patients. In our analysis, we progress from zeroth order through temporal informed statistics, both from an individual patient’s standpoint and also considering the collective effects. Our findings confirm our intuition that knowledge of common disease progressions results in higher predictability bounds than treating disease histories independently. We complement this result by showing the point at which the temporal dependence structure vanishes with increasing orders of the time-correlated statistic. Surprisingly, we also show that shuffling individual disease histories only marginally degrades the predictability bounds. This apparent contradiction with respect to the importance of time-ordered information is indicative of the complexities involved in capturing the health-care process and the difficulties associated with utilising this information in universal prediction algorithms.

Rising health care costs is an increasingly pressing societal issue. The reactive nature of medical care has implications on the ability to treat diseases because interventions are often only designed once symptoms have surfaced. Interventions to prevent the progression of chronic diseases before they emerge has the potential to reduce the burden on health care^1^. One main challenge in order to accomplish better medical care is the systematic management of patient data and its integration with medical knowledge bases to be able to take informed action, such as recommending a therapy or a diagnostic test^2^,^3^,^4^. Patient- and population-level data are crucial to advance evidence-based medicine.

Computing prediction scores calculated from health risk factors of developing incident coronary heart disease^5^,^6^, or the link between obesity and cancer^7^, are examples of statistical methods based on population data. Incorporating medically significant risk factors has been a major challenge because the improvement of risk prediction has to be assessed and quantified appropriately^8^. Data mining techniques^9^,^10^ and mathematical programming^11^ have been used to discover predictive rules from medical and biological data to improve disease prediction. In addition, artificial neural networks have been used to predict osteoporosis^12^ and cancer^13^. Genome-wide association studies, on the other hand, attempt to provide fundamental insights into disease risk factors, which can be explained by the genetic make-up of a patient and the resulting susceptibility to diseases. Recent advancements in human genome sequencing have broad implications permeating through several areas of biology including the discovery of genetic factors for common and rare diseases^14^,^15^,^16^. A number of studies have been conducted to utilise imperfect information in electronic health records (EHRs) to inform genome-wide association studies^17^, to correlate with physiological models of glucose variations^18^, or to identify multiple sclerosis patients and derive a clinically meaningful disease severity score^19^.

Despite these numerous efforts towards proactive medical care, it is still elusive how well EHRs can reliably inform clinical decision making. The challenges associated with EHRs are manifold, and established protocols are required in order to be able to apply clinical logic to recorded patient data^20^,^21^,^22^. Aligning clinical outcomes with EHRs that span multiple health care providers requires a rigorous data quality assessment framework that includes iterative improvements for individual care providers to correct potential errors^23^. Bailey *et al.* demonstrated that multi-institutional EHR data can be used successfully to monitor childhood obesity to complement national surveys^24^. Typically, the data of a patient’s recorded history is incomplete and sparse. Gaps may hide important information from clinical support systems which can lead to different interpretations of laboratory tests^25^. Additionally, differences in clinical workflows and associated data recordings are likely. Data accuracy is compromised by random and systematic errors, which are mainly due to the complexities involved in capturing the health care processes. For example the severity level of chronic diseases may not be captured, because only a subset of the diagnostic codes at institutions are actually used. Biases in coding practices of administrative data are also introduced because the amount of financial reimbursement is linked to the codes assigned to a given encounter^26^.

In this report we explore the degree to which health progression can be predicted given a patient’s historic record of diagnostic codes in EHRs. From a conceptual standpoint, the scope of our work is focused on studying the EHR as an object in itself^20^, and from a methodological standpoint, our approach is similar in character to looking for the limits of predictability in human mobility^27^. Several researchers used information-theoretic methods to analyse the ability to predict specific health conditions in EHRs^28^,^29^,^30^. However, it is still not well understood how well a predictive algorithm could serve in principle as diagnostic codes of EHRs are revealed one at a time without prior filtering. Our contribution is a quantitative assessment of predictability estimates from a large EHR dataset. Our approach relies on an extended formalism of predictability to leverage collective effects in sequences with a common alphabet.

Overall, we found that knowledge of time-correlated statistics results in increased predictability compared to first-order ones. We characterise how time-correlated information influences predictability in two ways. First, we analyse how knowledge of a window of *n* − 1 symbols of a patient’s disease history improves our ability to predict the *n*-th next disease as a function of *n*. From this, we discover that predictability is not significantly improved beyond a window of size 2. Second, we randomly permute the order of diseases within each patient in our database and find that our entropy and predictability statistics degrade only marginally. Surprisingly, this appears to contradict our statement that the temporal order of EHRs presents opportunities for prediction. However, this apparent contraction indicates that many disease patterns tend to involve alternate serialisations of the same set of codes. Yet, we are able to demonstrate that a significant predictive quality is present in the unordered expression of short sequences of diseases of the EHR compared to a worst-case baseline where correlations with a patient’s health state are artificially removed. We conclude that two principle aspects drive our results. First, EHRs are interspersed with relatively acute conditions that may not convey any information about its embedded history. Filtering these out, may improve the temporally correlated predictive power. And second, our EHR spans on average about 6.5 years, which may be too short to give rise to long-term temporal correlations of health progressions.

## Results

Our results are organised as follows. We study the estimates of the entropy rate *S* and the related predictability Π both from an individual’s and a collective perspective. At an individual level, each patient’s EHR is analysed independently, where the entropy rate estimates consider only the available information within each disease sequence. In contrast, at a collective level we impose a common alphabet across the EHR records which is in fact defined by the International Statistical Classification of Diseases and Related Health Problems edition 9 (ICD-9) coding scheme. Modelling transition systems given a common alphabet enables us to draw upon the main approaches and results of language modelling. Specifically, this means we compute *n*-grams of increasing order. In this context, we use the cross-entropy rate evaluated on a 10-fold cross validation dataset where in each fold 90% of the dataset is used to train the probabilistic model and 10% is used to assess the accuracy of the model. In our modelling assumptions we know that EHRs are not a stationary ergodic process. However, similar to the natural language literature^31^,^32^,^33^, we can assume to maintain stationarity, i.e., unchanging maximum likelihood estimates of the probabilities after having parsed sufficiently many disease sequences. In fact we provide empirical evidence in the “Methods” section that our estimators converged by varying the training set size from 70–90%. Each disease history is considered independent of others and we process those in randomised order.

From a methodological standpoint we follow Song *et al.* who introduced a notion of predictability that mathematically links entropy of a data source to an upper bound of predictability^27^. Noting that entropy is minimised by concentrating the probability mass of the most likely next symbol and assuming all other symbols are equally likely, the upper bound is calculated by solving 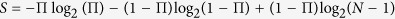 for Π given the entropy *S* and the number of symbols *N* in the alphabet (see the “Methods” section in the “lSupplemental Material” for more details). This definition of predictability is a theoretical construct that serves the purpose of illustrating that no algorithm exists that can do better than this estimate. Given the knowledge structures imposed by different entropy rate estimates, the resulting predictability bounds can inform an algorithmic designer on practical means of utilising information inherent in the data source. Accordingly, we can now plug in the entropy rate estimates to calculate upper bounds on predictability.

The entropy rate and corresponding predictability estimates are presented on three different levels of granularity. The ICD-9 coding scheme is organised in a hierarchy. The highest level of this hierarchy classifies the diseases into 19 categories (not shown in this article). The second highest level distinguishes 186 categories of diseases, followed by 1719 categories, and finally 12462 disease codes. This means we can impose different alphabets to assess whether higher-level categories result in more robust or better estimators. Our dataset comprises 516,276 patients with at least 20 and on average 31.16 diagnostic codes in their EHR.

### Individual Analysis

Specific entropy rate estimators are linked with an assumed knowledge representation. In our individual analysis we calculate three entropy statistics for each history *h*_*i*_: a) random entropy 
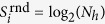
 which assumes no knowledge about the diseases and is equivalent to uniformly sampling from *N*_*h*_ distinct diagnostic codes present in history *h*; b) uncorrelated entropy 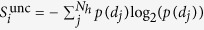 which is also known as Shannon’s entropy that incorporates the probability of each diagnostic code occurring; c) and finally correlated entropy 
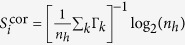
, where *n*_*h*_ is the length of medical history *h*_*i*_, and Γ_*k*_ is the length of the shortest subsequence of the medical history starting at position *k* and which does not previously appear from position 1 to *k* − 1^34^. In the order at which these estimators are presented they encapsulate more and more information about the sequences. *S*^cor^ can be thought of as our best estimator of the entropy rate *S* of the medical history. It is important to note that the correlated entropy is an efficient estimator in that it converges towards the true entropy value fast and needs relatively short sequences^34^. However, the individual disease sequences are not stationary ergodic and hence we need to view the corresponding results with some caution.

**Figure 1:**
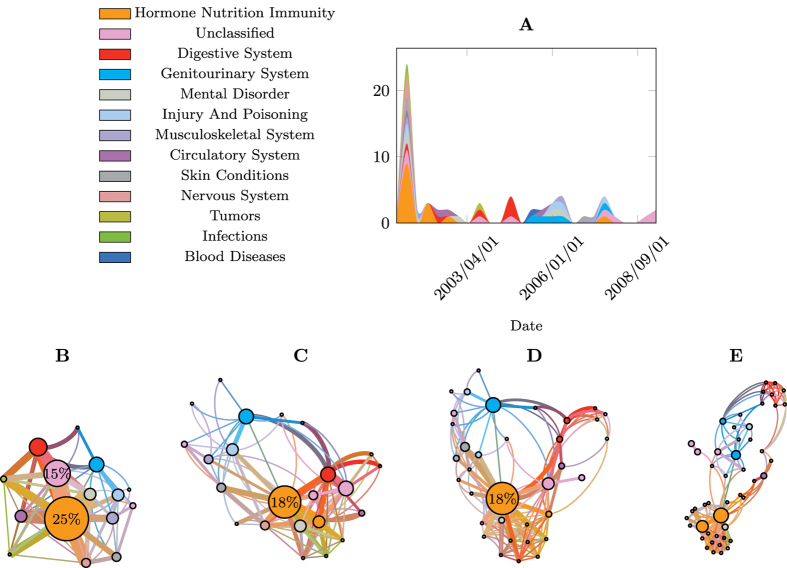
Medical history of one anonymised patient with 28 hospital visitations and 64 diagnoses over a 9 year period. (**A**) The personal disease history as plotted according to the top-level category of the ICD-9 classification scheme and aggregated for each quarter of a year. The most common diseases for this patient are related to hormone nutrition immunities, digestive, and genitourinary diseases. (**B**) visualises possible disease associations for the first level category of diseases. These disease associations are based on the chronological order of the personal disease history, where a connection between diseases is established if a set of diagnoses at at hospital visitation *t* + 1 follows a set of diagnoses at the previous hospital visitation. (**C**–**E**) provide successively more detail on the diagnostic code ranging from the second level category to the actual ICD-9 code.

For illustration, we begin by running our individual statistics on a random patient, and present the results in Fig. 1. This patient has been diagnosed with 48 distinct ICD-9 codes yielding 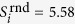, while accounting for the distribution of the diagnostic codes we get 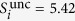. This means that if we assume that the patient’s medical history follows a uniformly random pattern, then any prediction scheme cannot guess the next diagnosis with better chance than 1/48 = 0.021. Accounting for the distributional characteristics of this patient’s medical history results in a value very close to the random entropy 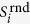, and does not improve the predictability substantially. However, considering the temporal order of the medical history, our entropy rate estimate of this patient’s medical history is 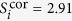. This means that ≈3 bits are necessary to encode the information of correlated medical histories for this patient, or the probability of predicting correctly the next disease code is 2^−2.91^ = 0.13. In other words, 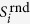 and 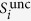 both indicate that each diagnosis in a health encounter produces an average of about 5.58 bits of new information, that is an average of about 2^5.58^ ≈ 48 possible next diagnostic codes. In contrast, a 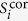 of about 3 bits indicates that the real uncertainty in a new diagnosis is about 2^3^ = 8 codes.

**Figure 2:**
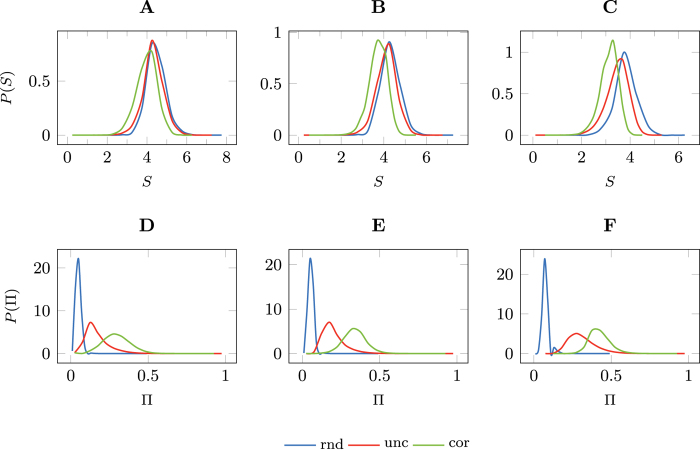
Entropy distributions of individual medical histories (first row) and upper bound on predictability (second row). The columns represent the entropy and predictability results for the different category-level views of the ICD-9 histories, starting from CAT_4_ through CAT_2_. Each curve represents a lens through which we view the data looking at zeroth-order (rnd), first-order (unc) to time-correlated statistics (cor).

Calculating these entropies over our entire patient cohort of over half a million people we obtain the results shown in Fig. 2, which displays the distribution of the three entropy statistics, 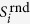, 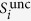, and 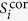 at the category-level view of the ICD-9 codes, shown in different subplots from A for CAT_4_ through C for CAT_2_ (see section Data Preliminaries in the “Supplemental Material” for more detail). For each category, note how the distributions of *S*^rnd^ and *S*^unc^ are virtually indistinguishable for the detailed ICD-9 code in Fig. 2A and their parent category in Fig. 2B, suggesting that the occurrence of ICD-9 diagnoses as characterised from the histogram of each sequence is practically uniform. In other words, by examining the EHR of a particular patient, most diagnoses in the sequence are given only once and very few exhibit counts of 2 or more, suggesting little hope for prediction schemes relying on distributional patterns to be successful. A separation between the uncorrelated entropy and the random entropy can only be observed for category CAT_2_ in Fig. 2C, corresponding to the second lowest (coarser) level of specificity of the ICD-9 hierarchy (for brevity of exposition we omit the lowest level of specificity). This separation occurs because more diagnostic codes are grouped together under equal categories and patterns of repetition begin to emerge, which results in lower entropy values of *S*^unc^. Also along the category variation, the distribution of *S*^cor^ exhibits significantly lower entropy values compared to both *S*^unc^ and *S*^rnd^ (P-value: < 0.001; one-sided Kolmogorov-Smirnov test), suggesting that knowledge of time-correlated events reduces the entropy of the symbol sequence.

Figure 2D–F show the corresponding predictability distributions of individual medical histories. We observe similar qualitative characteristics between the predictability curves here and the entropy counterparts in Fig. 2A–C, with a difference in scale due to the logarithmic nature of the entropy values. This highlights how minor differences in entropy result in more pronounced differences in predictability.

The predictability values derived from our random and uncorrelated entropy estimates have a mean of approximately 3% and 12% respectively, while time-correlated information further increases the upper bounds on the limits of predictability to 29% (pair-wise one-sided Kolmogorov-Smirnov test with P-value < 0.001). In fact using random entropy yields a lower bound on predictability with Π = 1/*N*, because we cannot do worse than selecting one of the *N* diseases with equal probabilities. The results indicate that from a predictability perspective, both distributional and time-correlated information have a significant impact on the ability to guess the next symbol in the disease history. As higher levels of the ICD-9 hierarchy are used to describe the health conditions better predictability is attained in lieu of less detail in phenotypic expression (i.e., a reduced alphabet of disease codes).

Concluding the individual analysis we found that the predictive quality is dominated by chance alone. E.g., the upper bound of predictability derived from time-correlated information is 29% given the complete ICD-9 coding scheme. As the disease codes are projected onto higher categorical levels this estimate improves to 43% for the second coarsest category. This means that in 57% of the cases we cannot do better than uniformly randomly guessing the next disease code. This result is not surprising since most disease progressions do not exhibit any periodic behaviour. Hence, we are not able to utilise the past in order to predict future diseases.

### Collective Analysis

Up to this point, our considerations on entropies and predictability were viewed through the lens of the individual patient. However, the crucial question remains of whether predictability can be pushed further utilising the combined knowledge of the entire patient population. Intuitively, while individuals may not exhibit repeating patterns within their own histories, a population-level scan through the EHR is going to reveal progression patterns that can be regarded as signatures that occur frequently in individual disease sequences. With enough patients exhibiting block-wise similar patterns transition models can be built that encode the conditional relationships between diseases. If one considers progressions of diagnostic codes while maintaining absolute time references, advances in clinical care, such as the introduction of new medical procedures, can result in significant differences in medical care before and after such events^35^. Such events give rise to non-stationary electronic health records. However, in our study we consider the time stamp with the objective of identifying the order of ICD-9 codes occurring in the EHR instead of modelling absolute time. As a consequence we lose the time reference to such events and we are less exposed to related non-stationary effects.

Given that all symbols in the disease sequences of our EHR belong to a common alphabet (see the “Methods” section in the “Supplemental Material” for a description of ICD-9 codes and the alphabet of our EHR dataset), we are able to regard each disease sequence as originating from the same “information source”, which is unlike the studies conducted on mobility^27^,^36^. In this context, we perform a collective analysis of the EHRs of all patients, which is similar to building language models for English text, for example^37^,^38^. In considering the collection of patients as a whole, the natural extension to the individual entropy analysis done above is to build *n*-gram models with increasing order *n* that extract knowledge about common progressions in the diagnosis sequences. With the knowledge of counts of *n*-grams, the conditional probability of the set of diseases as the most likely next disease in a sequence can be assessed. With large alphabets, higher order models face the challenge of sparsity in the language model. This means that progression patterns are becoming increasingly rare and as a consequence introduce a bias in the entropy rate estimates. We assess the information gain of higher-order models *M* using cross-entropy which is approximately unbiased, 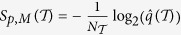, and perplexity, 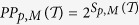, of a smoothed language model 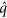 constructed using training data *T* and evaluated against a hold-out validation dataset *V* (see the “Methods” section in the “Supplemental Material” for more detail). Additionally, we evaluate how faithfully the probabilities are represented across the models produced during cross-validation. Small percentage changes in the joint *n*-gram probabilities indicate that we do not violate the stationarity assumptions. We find that 95% of the percentage changes between the joint probabilities of trigram models across the folds of cross-validation are less than 18.5% for the complete ICD-9 code. This estimate accounts for the relative frequencies of the *n*-grams. Larger percentage changes are generally due to rarer *n*-grams. Projections onto more general category levels reduce this estimate to less than 14.5% for the third category level and 5.5% for the second category level (see details in the section entitled “Assessing *n*-gram Quality” in the “Supplemental Material”). With this result we believe we are well within our stationarity constraints in the vast majority of the cases, especially considering the projections of the ICD-9 alphabet to more general category levels.

**Figure 3:**
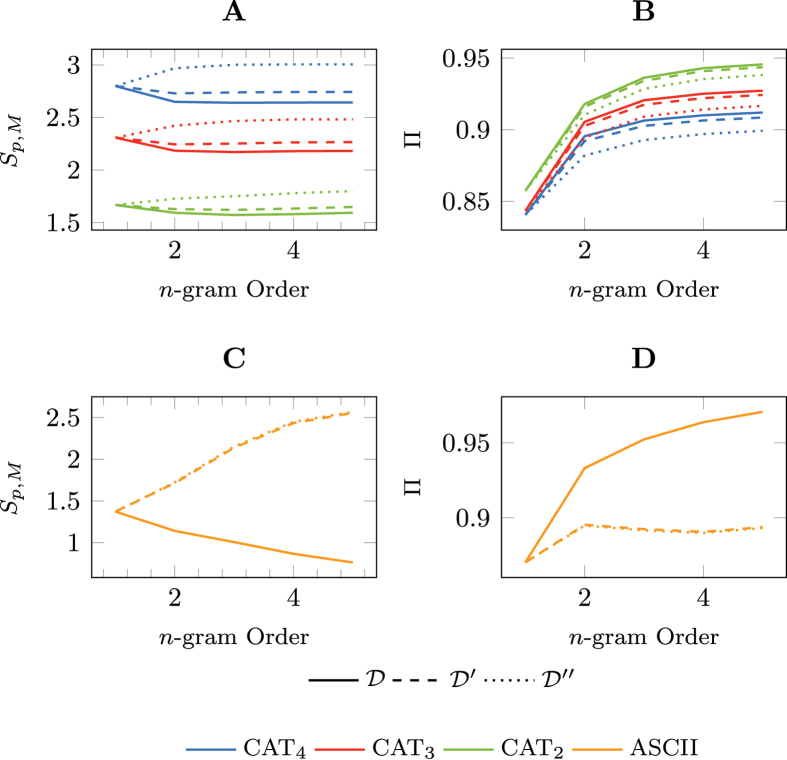
*n*-gram model cross-entropy and corresponding predictability bands of the hold-out validation dataset. The statistics are computed for the categories CAT_4_ through CAT_2_ and for the original dataset *D*, individual histories shuffled *D*′, and the entire dataset shuffled *D*″ (**A**,**B**) and for the Brown Corpus (**C**,**D**).

Figure 3A shows the cross-entropy rate estimates from the *n*-gram models with orders 1 ≤ *n* ≤ 5. As the time-correlated entropy measure suggests, increasing the *n*-gram order will decrease the entropy and improve upon the predictability. In fact, with an *n*-gram order of 2 the upper predictability bound exceeds the previous Π^cor^ = 29% by 61 percentage points to Π^cor^ = 90% (see Fig. 3). As a consequence, only 10% of predictability is due to chance alone. An improvement of the upper bound of predictability beyond the order of *n* = 2 can only be observed for coarser category levels. However, all pair-wise differences for *n* > 1 are statistically significant given the results of the 10-fold cross-validation.

Armed with the insight that a window of size 2 already captures most of the predictive power in our dataset, we further challenge the significance of time-ordered information on predictability. We do so by calculating the cross-entropy rate, *S*_*p,M*_ (*V* ′), using the same language model 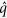 as before, validated against a hold-out sample *V* ′ of our dataset of shuffled individual histories *D*′. When we control for the within-patient order of diseases, we observe that the shuffled dataset shows a negligible reduction in the upper predictability bound at an *n*-gram order of 2 of 1 percentage point. Thus, the language model 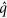 does almost equally as well in predicting the next disease whether the individual histories are shuffled or not.

This result presents an apparent contradiction for two reasons. At an individual level, we report that the uncorrelated entropy rate *S*^unc^ is significantly higher than the time-correlated entropy rate *S*^cor^. This indicates that the order of events in the various disease histories is important. And at a collective level, our *n*-gram analysis yields entropy rate estimates that become lower as the order *n* of the *n*-gram increases, once again indicating that knowledge of time-ordered events carries an important amount of the information content of the disease sequences. We are able to explain this effect and resolve such contradiction by noting that there is some symmetry in a patient’s disease history-it does not matter how one reads it. The disease patterns tend to involve alternate serialisations of the same set of codes, which was previously reported by Patnaik *et al.*^39^.

In order to obtain further insights into this finding, we introduce a second shuffling mechanism. We turn our dataset into disease sequences that are independent and identically distributed by shuffling our dataset such that we lose all within patient correlated disease sequences. Specifically, we repeatedly select two random disease sequences *h*_*i*_ and *h*_*j*_ and swap a randomly selected disease *d*_*i,k*_ in sequence *h*_*i*_ with a randomly selected disease *d*_*j,l*_ in sequence *h*_*j*_. After a large number of iterations we realise a dataset *D*″ that is effectively a full randomisation of the entire dataset. For this second shuffling method, we repeat the same calculations to obtain 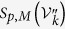, with respect to the original model 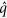 built from the original dataset *D*. In this case, we observe that the entropy and predictability statistics degrade significantly. This relative comparison leads us to the conclusion that a significant amount of predictability stems from the conditional expression of the diseases with a patient’s health state. However, the temporal order may be presented in many different serialisations.

If we compare these results to the predictability of natural language we begin to understand why predictive analytics on disease progressions is more challenging than that of English text for example. Intuitively, natural language is composed of a fixed set of words, which can be considered equivalent to disease progression patterns in the EHR dataset. We applied the same predictability analysis on the Brown corpus, which consists of over 1 million words in 51,763 sentences and over 6 million ASCII characters. The set of unique ASCII characters in this dataset has a cardinality of 77^40^. Shuffling the letter sequence within each sentence of the Brown corpus yields predictability curves that overlap exactly with the ones when shuffling letters across the entire corpus (see the second row in Fig. 3). This means that detecting spelling mistakes is very easy in English text, while for the EHR dataset the equivalent of a spelling mistake represents an alternate serialisation of the disease pattern.

## Discussion

The main purpose of this analysis is to quantify the predictability of a particular EHR dataset. Building upon a common coding scheme for diseases, namely the ICD-9 coding standard, we studied both individual and collective effects of over half a million patients. Our results show that knowledge of common disease progressions results in higher predictability bounds than treating disease histories independently. Harnessing knowledge of shared patterns across the population yields even higher predictability, but only up to a point, since the temporal dependence structure vanishes at an *n*-gram order of 2.

To gain further insight into the impact of collective intelligence and time correlations on predictability, we artificially altered the dataset in two ways. First, we shuffled the order of the diseases within each patient’s individual history. Surprisingly, our results show that shuffling at the patient level has little bearing on entropy and predictability statistics. This seems to indicate that the generating process of the EHR data is already so complex that the patterns observed in the data could just as well have been permuted. In other words, from an information-theoretic standpoint, one could encode the complete EHR just as efficiently if it were pre-processed through a stage of random permutation. Patnaik *et al.* arrived at similar results showing that many disease patterns tend to involve alternate serialisations of the same set of codes^39^. We can explain this result by noting that approximately 100 of the most probable diseases (out of approximately 12,500) absorb 50% of the probability mass, as shown in Fig. 3E in the “Supplemental Material”. Such concentration of mass is likely resulting in low-order patterns that are invariant when shuffled within a patient’s history *D*′.

Second, we shuffle all disease codes across the entire dataset, essentially randomising the entire EHR database. This time we observe that the predictability bounds do degrade markedly. With this result we establish that knowledge of a patient’s unique set of diseases, in the equivalent sense of a “bag of words”, bears a more significant impact on the predictability of the EHR, and the additional knowledge of the time-ordered diseases exhibits a negligible improvement. It is important to note that we did not remove any common or acute conditions that may not convey any information about its embedded history. In fact, EHRs are dominated by relatively acute diseases. Using the chronic condition indicator developed by the U.S. Agency for Healthcare Research and Quality (AHRQ)^41^ we find that 20% of the diseases in our database are chronic. Judiciously removing acute diseases has the potential to extract more pronounced chronic progressions.

Language models built on English text (e.g., the Brown corpus^40^) do not mirror this result. Instead, shuffling the characters of individual sentences results in predictability bounds that are very close to shuffling letters across the entire corpus. The difference between curating and analysing natural language and electronic medical records is that natural language (if spelled correctly) contains a fixed set of words, whereas the equivalent of words in disease histories does not follow spelling rules. Instead, the complexities of EHRs are a facet of data entry and associated workflows, the partial observability of health conditions, and of the health process itself.

In order to deal with some of the challenges associated with EHRs, tools have been developed to study the temporal information in medical records. Albers and Hripcsak used time-delayed mutual information to characterise the predictability of physiological time-series in light of irregularly and sparsely sampled data^28^,^29^. Their method uses predictability as a derived quantity in order to distinguish specific physiological models, e.g., glucose metabolism. High predictability can be attributed to a more complete physiologic time-series of sufficient length. This information can in turn be used to inform filtering techniques to carry out retrospective research on EHR data, where it is beneficial to include only highly predictable patients. Using time-delayed mutual information accounts for a varying degree of long-range correlations. Using *n*-gram models as we have done in our study does not accomplish comparable long-range correlations, because *n*-gram models estimate correlations among adjacent diagnostic codes in the EHR. In fact using our approach we found that the temporal dependence structure vanishes for orders higher than 2. This means that language models with a higher order do not impart any further information. An interesting extension of this work would be to look at all possible *n*-grams in a window *w* > *n* that allows gaps, which could recover meaningful temporal structures beyond the order of 2.

Perotte and Hripcsak also examined temporal aspects of individual disease documentations and demonstrated that relatively chronic conditions tend to have higher entropies whereas relatively acute conditions tend to have lower entropies^30^. The intuition behind this is that acute conditions are more isolated incidences, whereas chronic conditions do exhibit long-range patterns. This result indicates that the predictability of conditions is then inherently linked to their phenotypic expression pattern. Acute conditions tend to offer fewer opportunities to being predictable because of their spontaneous occurrences. We did not aim to distinguish between different classes of diseases to inform our predictability results. However, linking the predictability of a condition to an individual patient’s progression patterns is an interesting avenue of research.

Lastly, we establish a connection with the field of computational mechanics by noting that the key invariants of stochastic processes are: a) the information transmission rate or excess entropy, *E*, b) the statistical complexity, *C*, and, c) the per-symbol entropy rate, *S*, at which the process generates information-this last one being the same quantity that we estimate from the EHR database. One can obtain *S* given a process’s 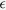-machine from the casual states’ priors and the transition matrix between them^42^. From this connection we note that our analysis only reveals a piece of the more complete and complex picture that computational mechanics could help us uncover. However, the goal of modelling the inner workings of the physical process that lies beneath our EHR database requires machinery to reconstruct the 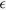-machine of the process. Algorithms have been developed, and one which would in principle be applicable to our data source is introduced in^43^. The time complexity of this algorithm scales as 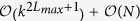, where *k* is the size of the alphabet and *L*_*max*_ is a parameter identifying the maximum length of the strings for which frequencies of their occurrence in the dataset of size *N* are collected. Assessing how this would translate to our dataset and thus being able to widen the scope of our study is an avenue of research that we are indeed interested in pursuing further.

In conclusion, we have presented another tool to study the predictive quality inherent in electronic medical records. Future work using combinations of more elaborate filtering techniques and a focus on specific conditions may have the potential to provide valuable insights into evidence-based clinical care.

## Additional Information

**How to cite this article**: Dahlem, D. *et al.* Predictability Bounds of Electronic Health Records. *Sci. Rep.* **5**, 11865; doi: 10.1038/srep11865 (2015).

## Supplementary Material

Supplementary Information
