# Supplementary material for: Predictability Bounds of Electronic Health Records
Source: Sci Rep. 2015 Jul 7;5:11865. doi: 10.1038/srep11865 (PMC4493571; doi:10.1038/srep11865)
Supplement: Supplementary Information [file srep11865-s1.pdf]

Supplemental Material – Predictability Bounds of Electronic Health Records

Dominik Dahlem[1,2,⋆], Diego Maniloff[2], and Carlo Ratti[2]

[1]IBM Research – Ireland, Dublin 15, Ireland
[2]Senseable City Lab, Massachusetts Institute of Technology, Cambridge, MA 02139, USA
[⋆]Correspondence to dominik.dahlem@gmail.com

Methods

Data Preliminaries

Data Source We examine anonymised electronic medical records collected over 10 years from January 2000 to July 2010 in the United States. The dataset consists of about 200 million visitation records which capture the health conditions of over 7 million patients. All data used for this study was collected privately and not specifically for research by the Medical Quality Improvement Consortium (MQIC) data warehouse. Data was contributed by users of the Centricity EMR electronic health record (GE Healthcare, Barrington, Ill., USA). All patient data is de-identified with no name or address or any other personal information apart from age, gender, and ethnicity.

Data Schema Our source dataset, $\mathcal{D}^{\text{orig}}$, contains medical records with the following fields: a) anonymised patient identifier, b) timestamp, and c) a string defining the International Statistical Classification of Diseases and Related Health Problems edition 9 (ICD-9) diagnostic code. The patient identifier is a unique integer value, the timestamp represents the number of days from a fixed reference date, and the diagnostic code is a symbol from the ICD-9 classification of diseases.

ICD-9 Codes Each record in dataset $\mathcal{D}^{\text{orig}}$ contains a code from the International Statistical Classification of Diseases and Related Health Problems (ICD), edition 9. The ICD-9 system organises the different disease codes into hierarchical groups. Figure 1 shows a portion of this hierarchy. Given this scheme, one can think of each diagnostic code (field 'c' in the dataset) as a leaf node in the ICD-9 tree.

Medical Histories In order to construct medical histories from the visitation records in dataset $\mathcal{D}^{\text{orig}}$, we first group all records by the patient identifier (field 'a'), and sort each sequence by timestamp (field 'b'). This results in individual disease sequences h_i for each patient i consisting of time-ordered ICD-9 codes d_j:

$$h_i =< d_1, d_2, \dots, d_n > \ . \tag{1}$$

We intentionally discard the absolute time reference and instead are only focusing on the order at which the disease codes are given in the EHR. Disregarding the absolute time, however, does

not facilitate the investigation of disruptive changes in medical care, such as the introduction of new medical guidelines. These advancements in medical care are situated at a moment in time that can give rise to different medical care patterns before and after such changes are introduced [1]. In our study, we are more interested in global patterns of transitions and as a consequence neglect these potentially non-stationary transitions. Apart from time, there are other dimensions present and available in our dataset such as gender, ethnicity, age, and geographies that we have left for future studies to explore in great detail.

Given the hierarchical nature of the ICD-9 system, each disease sequence h_i can be cast into four different views:

$$\begin{aligned}
\mathrm{CAT}_1(h_i) &= < \mathrm{CAT}_1(d_1), \mathrm{CAT}_1(d_2), \ldots, \mathrm{CAT}_1(d_n) >, \\
\mathrm{CAT}_2(h_i) &= < \mathrm{CAT}_2(d_1), \mathrm{CAT}_2(d_2), \ldots, \mathrm{CAT}_2(d_n) >, \\
\mathrm{CAT}_3(h_i) &= < \mathrm{CAT}_3(d_1), \mathrm{CAT}_3(d_3), \ldots, \mathrm{CAT}_3(d_n) >, \\
\mathrm{CAT}_4(h_i) &= < \mathrm{CAT}_4(d_1), \mathrm{CAT}_4(d_2), \ldots, \mathrm{CAT}_4(d_n) > = h_i\,.
\end{aligned}$$

where $\mathrm{CAT}_c(h_i)$ represents patient's i medical history as described by the c-th category level of the ICD-9 hierarchy (1 being the topmost category and 4 being the fully specified ICD-9 code). As an example, “Amoebic lung abscess” (code 006.4), has the following categorical decomposition (see figure 1 for reference):

CAT_1(006.4): Infectious and parasitic diseases

CAT_2(006.4): Intestinal infectious diseases

CAT_3(006.4): Amoebiasis

CAT_4(006.4): Amoebic lung abscess

Note how $\mathrm{CAT}_4(006.4) = 006.4$, and in general $\mathrm{CAT}_4(h_i) = h_i$.

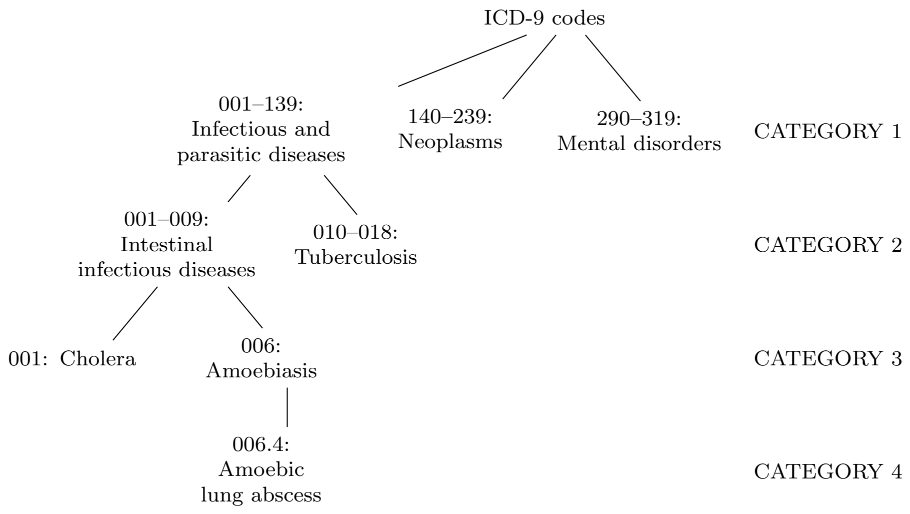

Figure 1: A portion of the ICD-9 tree. The right-hand side shows the categorical specification of a disease, whereby the high level categories (1&2) indicate the more general groupings of a disease, and the low level ones (3&4) provide greater specification.

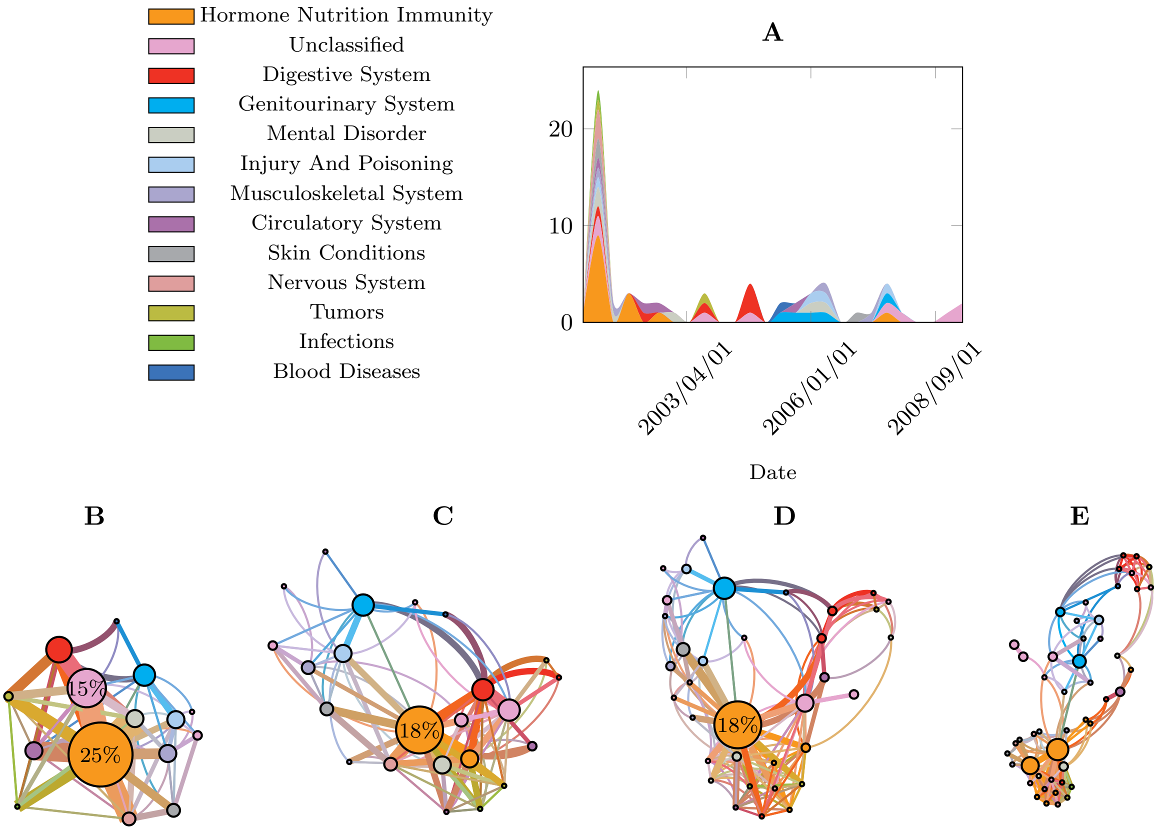

Figure 2: Medical history of one anonymised patient with 28 hospital visitations and 64 diagnoses over a 9 year period. **A** The personal disease history as plotted according to the top-level category of the ICD-9 classification scheme and aggregated for each quarter of a year. The most common diseases for this patient are related to hormone nutrition immunities, digestive, and genitourinary diseases. **B** visualises possible disease associations for the first level category of diseases. These disease associations are based on the chronological order of the personal disease history, where a connection between diseases is established if a set of diagnoses at at hospital visitation $t+1$ follows a set of diagnoses at the previous hospital visitation. **C** to **E** provide successively more detail on the diagnostic code ranging from the second level category to the actual ICD-9 code.

A Look at an Individual Patient In order to better grasp the different categorical views of a patient's medical history, figure 2 depicts the medical history of a randomly chosen patient who had been diagnosed with 64 medical conditions or symptoms on 28 hospital visitations. Because of the hierarchical classification scheme of ICD-9, we show 4 levels of detail of how diseases are associated with each other for the randomly chosen patient (see 1.1 for details on the ICD-9 hierarchy). Figure 2 **A** shows the quarterly summarised timeline of hospital visitations and the diagnosis count of the 13 disease categories the patient was diagnosed with. The disease history of this random patient can also be visualised in a network of disease associations, where we plot disease transitions in chronological order (figure 2 **B-E**). Any diagnosis recorded on a particular date t links to diagnoses recorded at the next hospital visitation at date $t+1$. The weight on the links corresponds to the number of times this transition has occurred, while the size of the node represents the prevalence of this particular diagnostic code in a patient's history. The coarsest level of detail is provided through 13 disease categories in figure 2 **B**. Diseases classified

under hormone nutrition immunity (such as Diabetes) make up 25% of this patient's diagnosed diseases followed by unclassified diseases (15%) and digestive diseases (9%). Figure 2 **C** and **D** provide more fine grained classifications and figure 2 **E** represents the actual 5 digit ICD-9 codes.

Descriptive Statistics and Filtering Rules Prompted by the challenges associated with EHR data [2], we calculated a variety of descriptive statistics, shown in figure 3. From these metrics, we introduced the following filtering rules to curtail certain artifacts in the dataset.

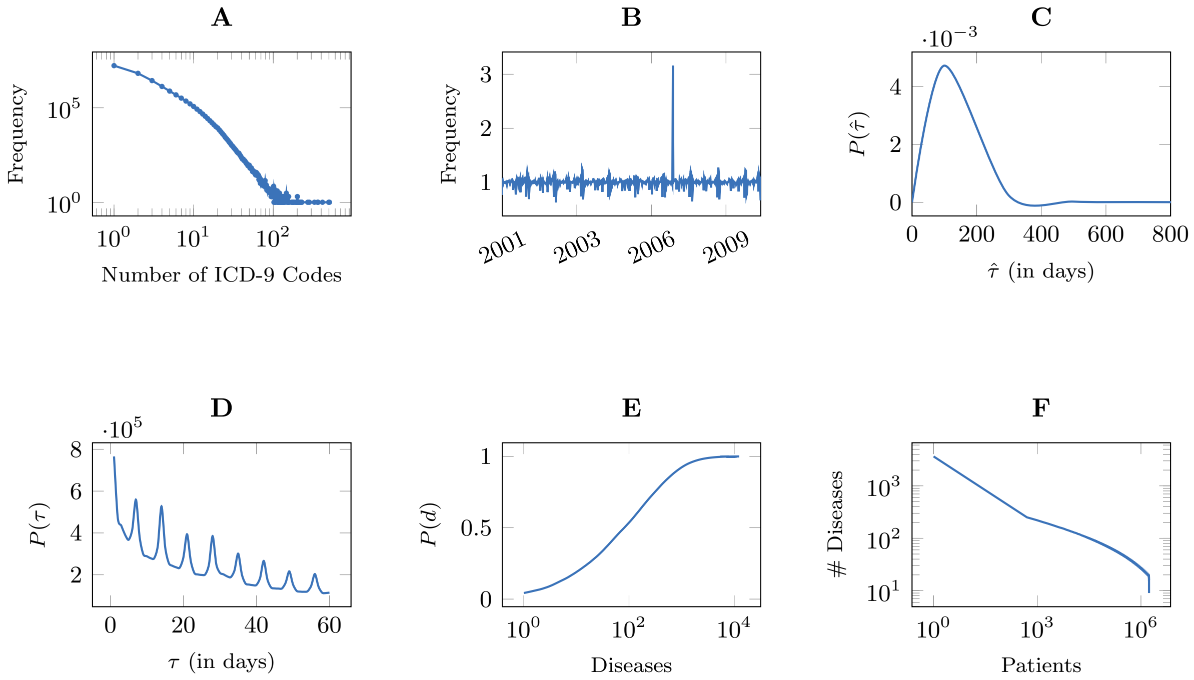

Figure 3: Descriptive Electronic Medical Record Statistics. **A** shows the frequency of the number of diagnoses associated with a single health encounter across the entire EHR. **B** shows the time-series of the number of visitations in each week from January 2001 to July 2010 normalised by the median of the corresponding month. **C** presents the distribution of the median inter-arrival times of each patient's health encounters. **D** presents the histogram of the inter-arrival times up to 60 days. **E** shows the cumulative probability distribution of the ICD-9 codes in the dataset. **F** presents the number of diagnostic codes found in a patient's EHR.

Figure 3 **A** shows the frequency of the number of diagnostic codes assigned to a health visitation across the entire EHR dataset (a health visitation event is identified by the value of the timestamp, field 'b', in the dataset). While it is common practice to record more than one diagnostic code per health visitation, we found several instances where tens or even hundreds of codes were associated to a single patient on a single day (these batch entries likely stem from data imports). Figure 3 **B** gives further evidence to the presence of batch entries, where a single day receives an unusual number of diagnoses. For this reason, we removed all encounters that resulted in more than two diagnostic codes, with the caveat that such a cutoff may exclude patients with complex medical conditions diagnosed on a single health encounter.

The distribution of the median inter-arrival time of diagnoses for the patients is shown in figure 3 **C**. The median inter-arrival is on average 100 days between health encounters. Interestingly, figure 3 **D** illustrates some of the characteristics of health encounters in that they are typically scheduled in multiples of 7 days for follow-up visits. Figure 3 **E** shows

the cumulative probability distribution of the disease codes occurring in the EHR dataset and figure 3 **F** gives the count of diagnostic codes per patient. One can observe that there is a large number of patients that have had only 1 or 2 visits recorded in the system, which can be for a number of reasons, ranging from good health to switching to other health care providers. For this reason, we removed all patients that have a relatively short medical history of less than 20 diagnostic codes. Other researchers used different techniques to filter their dataset, for example by removing the most frequent diseases [3]. This can have the benefit that progression patterns are not dominated by common diseases that are independent and identically distributed.

Applying these two filtering rules resulted in a reduced dataset $\mathcal{D}$ of 516,276 patients with an average medical history length of 31.16 and the average history spans 6 years and 5 months.

Information-theoretic Background The simplest and most intuitive way of characterising the predictability inherent in EHRs is to invoke an information-theoretic approach to quantify the amount of information in the disease sequences. Thus, we set out to estimate entropy rates for each individual disease history h_i, and then move on to estimate the entropy rate of the entire dataset as a collection of disease sequences.

We model each disease history, h_i, as a sequence of discrete random variables $\{D_i\}$. Depending on which category level of the ICD-9 hierarchy we focus on, each D_i will vary over the following alphabet:

for category 1 (CAT_1), the alphabet is the set of first-level categories in the ICD-9 hierarchy, whose size in our dataset is 19 symbols;

for category 2 (CAT_2), the alphabet is the set of second-level categories in the ICD-9 hierarchy, whose size in our dataset is 186 symbols;

for category 3 (CAT_3), the alphabet is the set of third-level categories in the ICD-9 hierarchy, whose size in our dataset is 1719 symbols;

for category 4 (CAT_4), the alphabet is the entire set of ICD-9 codes, whose size in our dataset is 12462 symbols.

In what follows, unless otherwise specified, we will refer to our analysis at the fourth-level category. Our initial analysis follows the approach taken by Song et al. to explore the predictability of human mobility [4].

The entropy of a single random variable is a measure of its uncertainty, and the entropy rate of a sequence of n random variables is the per-symbol entropy of the n random variables [5]. Given a sequence of diagnostic codes, $\{D_i\}$, the entropy rate S of $\{D_i\}$ is a parameter that quantifies the average amount of information that is produced by each symbol [5, 6]. Intuitively, it is a measure of how "unexpected" each new symbol is as we read the sequence.

Entropy Calculations Given a symbol sequence $h =< d_1, d_2, \ldots, d_n >$, we estimate its entropy rate via a series of approximations $S(0)$, $S(1)$, ..., which successively take more of the statistics of the sequence into account [7]. We refer to $S(n)$ as the entropy estimate that measures the amount of information due to statistics extending over n successive symbols in h.

$S(n)$ is calculated as:

$$S(0) = \log_2(N_h), \tag{2}$$

$$\begin{aligned} S(n) &= S(d_j|\mathbf{b}_i) \\ &= -\sum_{i,j} p(\langle \mathbf{b}_i, d_j \rangle) \log_2[p(d_j|\mathbf{b}_i)] \qquad (3) \\ &= -\sum_{i,j} p(\langle \mathbf{b}_i, d_j \rangle) \log_2[p(\langle \mathbf{b}_i, d_j \rangle)] \\ &\quad + \sum_i p(\mathbf{b}_i) \log_2[p(\mathbf{b}_i)],\ n \geq 1\,, \qquad (4) \end{aligned}$$

where N_h is the number of different symbols in h, d_j is a single target symbol, and $\mathbf{b}_i = \mathbf{d}_{i-n+1}^{i-1}$ is a block of the $n-1$ preceding symbols of d_j, i.e., the symbols from position $i-n+1$ to position $i-1$. As n increases, $S(n)$ includes more of the statistical structure of the sequence and the entropy rate S of h results as the limit value of $S(n)$:

$$S = \lim_{n\to\infty} S(n)\,. \tag{5}$$

An alternative definition of entropy is the quantity

$$S' = \lim_{n\to\infty} \frac{1}{n} S(d_1, d_2, \ldots, d_n)\,, \tag{6}$$

which measures the per symbol entropy instead of the conditional entropy of the last random variable given the past. Under the assumption of stationarity, both limits S and S' exist and are equal [5].

In order to assess the information content of a single medical history h, we focus on three statistics with important semantics [4] that arise from the progression of estimates of equations 2 - 4:

S_h^{rnd} Is the entropy of the history under the assumption that the diagnostic code distribution is uniformly random. This quantity is calculated as

$$S_h^{\text{rnd}} = S(0) = \log_2(N_h)\,,$$

where N_h is the number of distinct diagnoses in history h.

S_h^{unc} Is the entropy of the history considering the actual distribution of diagnosis codes in it. This quantity is calculated as

$$S_h^{\text{unc}} = S(1) = -\sum_j^{N_h} p(d_j)\log_2(p(d_j))\,,$$

where d_j is a symbol in h.

S_h^{cor} Is the entropy of the history considering the order in which diagnoses appear. This quantity is calculated as

$$S_h^{\text{cor}} = \left[\frac{1}{n_h}\sum_k \Gamma_k\right]^{-1} \log_2(n_h) \approx S\,,$$

where n_h is the length of medical history h, and Γ_k is the length of the shortest substring starting at position k and which does not previously appear from position 1 to $k-1$ [8]. S^{cor} can be thought of as our best estimator of the entropy rate S of the medical history.

Another way to think about these entropy estimates is that the random, S_h^{rnd}, and the uncorrelated entropy, S_h^{unc} disregard the time dimension. For the former entropy measure we only need the number of distinct symbols, and for the latter we require the frequencies of each symbol. Both are equal when the probability of each symbol is equally likely. The correlated entropy rate estimate, S_h^{cor}, on the other hand, models a medical history as a sequence of random variables. S_h^{cor} is known to be an efficient estimator that converges rapidly to the true entropy rate, even with quite short sequences. More specifically, the modelling assumption of estimator S_h^{cor} is that the medical history is a stationary ergodic process with real entropy rate $S > 0$ [8].

When analysing a group of patients that share certain traits (e.g., demographics, predisposition to certain diseases, etc.), the analysis of the information content of their medical histories should regard each sequence in the set as originating from the same "information source", such that we can extract knowledge about common health progressions occurring within the group of patients.

n-gram models [9] are a natural extension to the individual entropy analysis of the preceding section [7], and they provide estimates of the probabilities required by equations 2 – 4, taking into account the collective statistics of a group of medical histories. First, let us consider the general case of computing the probability of a disease history, h, composed of the diseases $d_1, d_2, \ldots, d_n$. Without loss of generality the probability $p(h)$ can be computed using the chain-rule:

$$p(h) = p(d_1)p(d_2|d_1)p(d_3|d_1d_2)\cdots p(d_n|d_1\ldots d_{n-1}) = \Pi_{i=1}^{n} p(d_i|d_1\ldots d_{i-1}). \tag{7}$$

For bigram models ($n = 2$), the probability of the history $h \sim p(h)$ is approximated by only taking the identity of the immediately preceding disease into account:

$$p(h) = \Pi_{i=1}^{n} p(d_i|d_1\ldots d_{i-1}) \approx \Pi_{i=1}^{n} p(d_i|d_{i-1}). \tag{8}$$

For $i = 1$ we introduce an artificial token to mark the beginning of a history in order to calculate the conditional probability $p(d_i|d_{i-1})$. Similarly, an end of history token needs to be introduced in order for the probability over all histories to sum to 1. The conditional probabilities in equation 8 are estimated using maximum likelihood estimation, which is simply the count of the bigram in the text divided by a normalisation constant amounting to the sum of the counts of all bigrams. For n-gram orders larger than 2 the probability of a history readily generalises to

$$\hat{q}(h) \approx \Pi_{i=1}^{n+1} q(d_i|\mathbf{b}_i), \tag{9}$$

where $\mathbf{b}_i$ is the block of diseases preceding d_i as before and the approximation is due to the finiteness of the n-gram order. As previously stated with the bigram model, the sequences are appropriately padded. Let $c(\mathbf{d}_{i-n+1}^{i})$ denote the number of times the n-gram occurred in the disease histories. Then the maximum likelihood estimates of $p(d_i|\mathbf{b}_i)$ are

$$q(d_i|\mathbf{b}_i) = \frac{c(\mathbf{d}_{i-n+1}^{i})}{\sum_{d_i} c(\mathbf{d}_{i-n+1}^{i})}. \tag{10}$$

Typically datasets are sparse, which limits the collection of sufficient statistics to reliably estimate the probability distributions. A consequence of this is that the counts $c(\mathbf{d}_{i-n+1}^{i})$ can be zero and hence lead to underestimates of $q(h) = 0$. Smoothing is a central issue in language modelling and is used to address this problem by assigning non-zero probabilities to these sequences [10]. Specifically, smoothing shifts some of the probability mass from high probabilities to low probabilities. Whenever, maximum likelihood estimates result from few counts, smoothing has the potential to improve the quality and accuracy of n-gram models significantly.

One of the simplest smoothing techniques used in practice is additive smoothing, which adds $0 < \delta \leq 1$ to the counts of the n-grams, yielding

$$q(d_i|\mathbf{d}_{i-n+1}^{i-1}) = \frac{\delta + c(\mathbf{d}_{i-n+1}^{i})}{\delta|V| + \sum_{d_i} c(\mathbf{d}_{i-n+1}^{i})}, \tag{11}$$

where $|V|$ is the vocabulary, which corresponds in our case to the different category levels of the ICD-9 coding scheme. We refer to Chen et al. who review and empirically evaluate several smoothing techniques in the domain of language modelling [10]. We employ Witten-Bell smoothing on our models [11], which interpolates the maximum likelihood estimate of the n-th order with the smoothed estimate of the $(n-1)$th order.

Cross-Entropy In general we define cross entropy of a random variable X with the true probability distribution p and a model M that stipulates an estimate $\hat{q}$ of that probability distribution as [12, 9]:

$$S_{p,M}(X) = S(X) + KL(p \parallel \hat{q}) = -\sum_{x \in X} p(x) \log [\hat{q}(x)], \tag{12}$$

where KL is the Kullback-Leibler divergence or relative entropy from $\hat{q}$ to p. $KL(p \parallel \hat{q}) = \sum_x p(h) \log (\frac{p(x)}{\hat{q}(x)})$ accounts for the model mismatch (in bits) onto the true distribution of X. The cross-entropy $S_{p,M}(X)$ quantifies the average length of bits needed to encode X sampled from the true distribution p with a coding scheme based on a language model $\hat{q}$.

It is a well-known fact that $S_{p,M}(X) \geq S(X)$, because the Kullback-Leibler divergence KL is always non-negative [5]. The better the model $\hat{q}$ is, the tighter the upper bound. Thus, it is important to note that given a particular model of X that approximates the true probability distribution p allows us to obtain an upper bound on the actual entropy [12].

This definition of cross-entropy is easily extended to define the cross-entropy of a stochastic process. Similarly to defining entropy rate in equation 6 as the limit of the entropy of a block of diseases as the size of the block approaches infinity, the cross-entropy rate of a stochastic process (here disease sequences) is defined as:

$$S_{p,M}(\mathcal{D}) = \lim_{n \to \infty} \frac{1}{n} S_{p,M}(X_1^n) = -\lim_{n \to \infty} \frac{1}{n} \sum_{(x_1,\cdots,x_n)} p(x_1^n) \log (\hat{q}(x_1^n)), \tag{13}$$

where X_1^n denotes $X_1, X_2, \ldots, X_n$ and $\hat{q}$ is the probability distribution over disease sequences estimated according to the language model given above.

Note that the true distribution of the disease histories is used once to generate the disease histories $\mathcal{D}$ and once to compute the probability $p(\mathcal{D})$. In our study we do not assume to know p, but instead evaluate the EHR as an object in itself and we do not claim that the results are universal. Nevertheless, we can model $\mathcal{D} = \{X_i\}$ as a stationary ergodic process and then the Asymptotic Equipartition Property (AEP) should hold [5]. By AEP,

$$-\frac{1}{n} \log [\hat{q}(x_1^n)] \to -\lim_{n \to \infty} \frac{1}{n} \sum_{(x_1,\cdots,x_n)} p(x_1^n) \log (\hat{q}(x_1^n)) \quad \text{as} \quad n \to \infty \tag{14}$$

$$= S_{p,M}(\mathcal{D}). \tag{15}$$

So, the cross-entropy $S_{p,M}(\mathcal{D})$ can be approximated by $-\frac{1}{n} \log [\hat{q}(x_1^n)]$ for sufficiently long disease sequences.

The inequality between the entropy and the cross-entropy of a random variable continues to hold for the entropy rate of a stochastic process $\mathcal{D}$

$$S_{p,M}(\mathcal{D}) \geq S', \tag{16}$$

and hence by estimating $S_{p,M}(\mathcal{D})$ we have an upper bound on the true entropy S'.

Bias Estimation Any entropy estimator, including the maximum likelihood estimator, will be subject to bias [13]. Intuitively, this is because the number of different possible blocks of length n increases exponentially with n and so does the necessary minimum length N of the sample sequence if one wants to determine the probabilities p faithfully [14]. The cross-entropy accounts for the bias through the Kullback-Leibler divergence from $\hat{q}$ to p (see equation 12). The Kullback-Leibler divergence can be interpreted as the information loss when using $\hat{q}$ to approximate the true (unknown) probability distribution p.

We can estimate the bias incorporated in the cross-entropy explicitly by following the same approach as Paninski and Miller [13, 15]. The Miller-Madow approach is an analytical bias correction that is derived from a Taylor expansion of the Kullback-Leibler divergence about the true underlying probability distribution p:

$$\mathrm{B}(S_{\hat{q}}) = S(\mathcal{D}) - S_{\hat{q}}(\mathcal{D}) = \frac{\hat{m} - 1}{2N} + \mathcal{O}(N^{-2}), \tag{17}$$

where $\hat{m}$ is an estimate of the alphabet size and N is the size of the EHR dataset. Figure 4 presents the biases normalised by the n-gram order for 3 different ICD-9 categories for each n-gram order. As the alphabet size increases with the sample size N fixed, we observe an increase in the corresponding biases. However, the classical asymptotics break down when $N \sim \hat{m}$ and as a consequence the bias is likely not fully accounted for [13]. We estimated $\hat{q}$ on the entire dataset with $N = 16,085,433$. Figure 4 **A** also shows the higher n-gram orders for which the bias is likely underestimated (the dashed lines). For the complete ICD-9 code specification n-gram with order $n \geq 3$ exhibit $N \sim \hat{m}$. As the disease codes are projected onto higher ICD-9 categories the order n for which this is the case becomes larger.

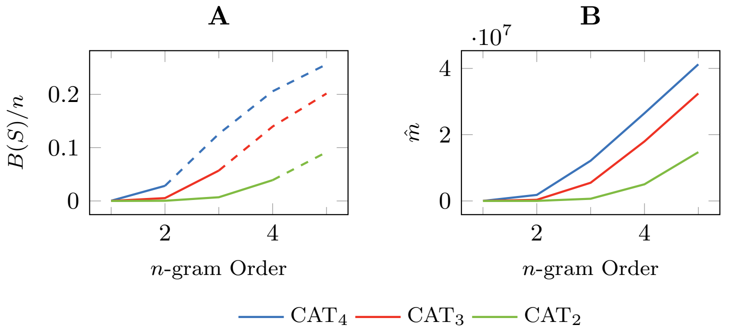

Figure 4: Normalised bias and alphabet size estimation of the Entropy estimates. For increasing ICD-9 categories the bias is presented in **A** and the corresponding alphabet sizes $\hat{m}$ are given in **B**. The dashed lines in figure **A** indicate that the sample size is on the order of the alphabet size, $N \sim \hat{m}$, which is where the Miller-Madow bias is likely underestimated.

Assessing n-gram Quality Large-scale language modelling, especially those with large alphabets or high orders or both, require a careful assessment of the underlying quality of the

model. As higher orders are being modelled more and more of the idiosyncrasies of the data source are embedded in the model and consequently n-grams may not improve upon the information content of the previous order. Since the true language model is not known, an upper bound to the entropy S' can be obtained from an approximation to p.

A common approach is to approximate the true language model is to perform an intrinsic model evaluation and compute the probability that the model assigns to a hold-out validation dataset $\mathcal{V}$ [9] using 10-fold cross-validation. From this probability the metrics cross-entropy and perplexity are calculated. We assess the quality of the trained n-gram model of medical histories by repeatedly drawing two subsets from our dataset without replacement, one for training (90%) and the remaining 10% for validation. From the training set, we build n-gram models of increasing order. These models provide the smoothed maximum likelihood estimates for the probabilities $q(d_i|\mathbf{b}_i)$. The validation set $\mathcal{V}$ is composed of disease histories $(h_1, h_2, \ldots, h_{l_\mathcal{T}})$. The probability of the validation set is then computed by

$$\hat{q}(\mathcal{V}) = \Pi_{i=1}^{l_\mathcal{V}} \hat{q}(h_i). \tag{18}$$

The cross-entropy of a model $\hat{q}$ on the validation set is calculating using $S_{p,M}(\mathcal{V})$ from equation 15. The cross-entropy $S_{p,M}(\mathcal{V})$ is an upper bound on the entropy S' [12]. Recall our previous notion of entropy as a measure of surprise or uncertainty about a data source. Cross-entropy quantifies the degree to which the test language departs from our smoothed model $\hat{q}$. A lower model cross-entropy tends to lead to a better performance in application scenarios. However, this does not need to be the case if one considers not only the probability mass but also the relative ordering. Language models tend to be optimised on perplexity, which is simply

$$PP_{\hat{q}}(\mathcal{T}) = 2^{S_{p,M}(\mathcal{T})}. \tag{19}$$

Using perplexity we can identify whether higher order n-gram models yield a significant improvement in prediction performance.

Additionally to assessing the n-gram quality by estimating cross-entropy or perplexity, we need to ensure that the entropies being calculated are consistent and converge. Because most of the above derivation of the modelling approach relies on stationary ergodic assumptions we may leave ourselves vulnerable to estimators that exhibit unstable behaviours. In order to elucidate whether this is the case, we sample differently sized training sets for our models, i.e., at 70%, 80%, and 90% using 10-fold cross-validation. Plotting statistical summaries across the folds for each training set size and n-gram order and type of dataset provides us with visual clues of the robustness of the entropy estimators. Figure 5 presents these results for the complete ICD-9 code specification of our EHR dataset. For brevity we focus on the most granular level of encoding the disease sequences, because higher level categories project disease sequences onto a smaller alphabet and hence are more robust. Indeed the results in figure 5 demonstrate that we get robust entropy rate estimators with an inter-quartile range of at most 0.005 bits per symbol across the folds and reductions of the same order as we increase the training set. Only our cross-entropy rate estimates of the population-wide shuffled dataset $\mathcal{D}''$ exhibits a slightly increasing trend as the sample size is increased.

Further to the above diagnostics, we evaluate the percentage change between the joint n-gram probabilities $q(\mathbf{b}_i)$ across the folds of our 10-fold cross-validation. We approximately meet our stationarity assumption when these percentage changes are very low and as a consequence we can claim that the probabilities are captured faithfully across the different training samples during cross-validation. Figure 6 presents the results for the full ICD-9 alphabet (figure 6 **A**), the third category level (figure 6 **B**), and the second category level (figure 6 **C**). The percentage changes in the unigram models are negligible. For the trigram models we find that 95% of all

percentage changes are smaller than 18.5%, 14.5%, and 5.5% for the respective alphabets. For the 5-gram models these changes are 21.5%, 18.5%, and 16.5% respectively. These statistics account for the relative frequencies of the n-grams.

Importantly, the larger the percentage change of a joint probability $q(\mathbf{b}_i)$ across the folds, the rarer the n-gram $\mathbf{b}_i$. Intuitively, this can be explained by the fact that observing a block of diseases only a few times in one fold can be exposed to more dramatic changes in the transitions across different samples of the cross-validation. As we increase the order of the n-gram models, more and more contexts exhibit low probabilities of occurring and hence the stationarity assumptions need to be reviewed carefully. Nevertheless, we can claim that our entropy rate estimators represent converged statistics and we are therefore not exposed to violations of the stationary ergodic assumptions of our approach, especially for n-gram orders smaller or equal to three.

Predictability Calculations We begin by defining p^{ML}, the probability that a patient will be diagnosed with the most likely next diagnosis. We then define predictability, π, as the weighted sum of p^{ML} over all possible medical histories. Finally, we show how to calculate an upper bound, Π, on predictability.

$p^{ML}(h_{n-1})$ is defined as the probability that the patient will be diagnosed with the most likely next diagnosis given a history of diseases $h_{n-1} =< d_1, d_2, \ldots, d_{n-1} >$:

$$p^{ML}(h_{n-1}) = \arg\max_{d} p(d_n = d|h_{n-1})\,.$$

In this way, p^{ML} takes into account the entire statistical structure of the patient's history up to time $n-1$.

The predictability $\pi(n)$ of a given medical history is defined as the best success rate to predict a patient's n-th diagnosis. We calculate it as the weighted sum of p^{ML} over all possible health trajectories up to $n-1$:

$$\pi(n) = \sum_{h_{n-1}} p(h_{n-1}) p^{ML}(h_{n-1}). \qquad (20)$$

Taking the limit as the length of the medical history increases yields the overall average rate with which we can successfully predict a patient's next diagnosis:

$$\pi = \lim_{n\to\infty} \frac{1}{n} \sum_{i} \pi(i). \qquad (21)$$

We want to relate the estimated entropies to a notion of predictability. An upper bound, Π, on π, can be derived as follows [4]: a) first, we use Fano's inequality to build a function S^F that is an upper bound of $S(d_n|h_{n-1})$; b) we then use S^F to derive an upper bound on the conditional entropy $S(n)$ of equation 3; and finally c) we extend the bound to the limit case to obtain $S \le S^F(\pi)$.

a) construct an entropy function S^F such that $S(d_n|h_{n-1}) \le S^F(p^{ML}(h_{n-1}))$, i.e., S^F is an

upper bound on the entropy of the n-th diagnosis random variable d_n:

$$
\begin{aligned}
d_n &\sim p(d_n|h_{n-1}) \\
d'_n &\sim \left(p^{ML}(h_{n-1}), \frac{1-p^{ML}(h_{n-1})}{N_h-1}, \ldots, \frac{1-p^{ML}(h_{n-1})}{N_h-1}\right) \\
S(d'_n|h_{n-1}) &= -p^{ML}(h_{n-1})\log_2 p^{ML}(h_{n-1}) \\
&\quad - (1-p^{ML}(h_{n-1}))\log_2(\frac{1-p^{ML}(h_{n-1})}{N_h-1}) \\
&= S^F(p^{ML}(h_{n-1}))\,.
\end{aligned} \tag{22}
$$

In this step we concentrate the probability mass at the most likely diagnosis code and assign equal probabilities to the remaining ones.

b) the weighted sum of $S(d_n|h_{n-1})$ over all possible medical histories h_{n-1} is just the conditional entropy $S(n)$ we defined in equation 3. We can use S^F to bound such a sum:

$$
\begin{aligned}
S(n) &= \sum_{h_{n-1}} p(h_{n-1})S(d_n|h_{n-1}) \\
&\le \sum_{h_{n-1}} p(h_{n-1})S^F(p^{ML}(h_{n-1})) \\
&\le S^F\left(\sum_{h_{n-1}} p(h_{n-1})p^{ML}(h_{n-1})\right) \\
&= S^F(\pi(n))\,.
\end{aligned}
$$

c) we can now derive the desired relation for S:

$$
\begin{aligned}
S &= \lim_{n\to\infty}\frac{1}{n}\sum_i S(i) \\
&\le \lim_{n\to\infty}\frac{1}{n}\sum_i S^F(\pi(i)) \\
&\le S^F\left(\lim_{n\to\infty}\frac{1}{n}\sum_i \pi(i)\right) \\
&= S^F(\pi)\,,
\end{aligned} \tag{23}
$$

where equation 23 is an application of the chain rule to the alternative definition of entropy presented in equation 6.

We are now ready to define Π as the upper bound on predictability. We calculate Π by plugging in an entropy rate estimate S and the number of distinct diagnoses N into $S = S^F(\Pi)$, and then solving for Π. Given that $S = S^F(\Pi) \le S^F(\pi)$, and that $S^F(\pi)$ monotonically decreases with π, we obtain $\Pi \ge \pi$.

Research Protocol In the following we are highlighting the research protocol that governs our findings. As mentioned above we use 10-fold cross-validation for all our statistics. In each fold k we use a training set $\mathcal{T}_k$ of 90% of the original dataset $\mathcal{D}$ to build our language model. Let us call this model $\hat{q}_k$ for each fold k. The remaining 10% of each fold make up

a hold-out validation set $\mathcal{V}_k$ over which we compute the cross-entropy rates and derive the upper bound of predictability. In parallel we permute the dataset $\mathcal{D}$ in two ways for each fold. First, we permute the order of the diseases for each patient in $\mathcal{D}$ independently in each fold to get a new dataset $\mathcal{D}'_k$. In order to exemplify this process, let us consider a patient with the following disease history: AABCCA. A possible permutation of this patient in fold 1 may result in BCCAAA and in fold 2 CCAAAB. Second, we permute diseases across all histories $\mathcal{D}''_k$, essentially removing any correlation structure in the diseases a patient might have had in his EHR. We sample 10% validation sets $\mathcal{V}'_k$ and $\mathcal{V}''_k$ without replacement of both permuted dataset respectively and compute the cross-entropy rates $S_{p,M}(\mathcal{V}'_k)$ and $S_{p,M}(\mathcal{V}''_k)$ using the model $\hat{q}_k$ we built on the original training set $\mathcal{T}_k$. Using the statistics for each fold we compute the 95% confidence intervals and perform one-sided statistical significance tests to quantify any differences.

The principal reason for permuting is to test whether we can claim any predictive quality in our dataset at all. If the entropy and corresponding predictability statistics do not degrade substantially for any of the permuted sets, then we cannot claim any predictive quality.

References

[1] Pivovarov, R., Albers, D. J., Hripcsak, G., Sepulveda, J. L. & Elhadad, N. Temporal trends of hemoglobin a1c testing. *Journal of the American Medical Informatics Association : JAMIA* **21**, 1038–1044 (2014).

[2] Hripcsak, G. & Albers, D. J. Next-generation phenotyping of electronic health records. *Journal of the American Medical Informatics Association* **20**, 117–121 (2013).

[3] Patnaik, D. *et al.* Experiences with Mining Temporal Event Sequences from Electronic Medical Records: Initial Successes and Some Challenges. In *Proceedings of the 17th ACM SIGKDD International Conference on Knowledge Discovery and Data Mining*, KDD '11, 360–368 (ACM, New York, NY, USA, 2011).

[4] Song, C., Qu, Z., Blumm, N. & Barabási, A.-L. Limits of Predictability in Human Mobility. *Science* **327**, 1018–1021 (2010).

[5] Cover, T. M. & Thomas, J. A. *Elements of Information Theory 2nd Edition (Wiley Series in Telecommunications and Signal Processing)* (Wiley-Interscience, 2006), 2 edn.

[6] Shannon, C. E. *The mathematical theory of communication* (University of Illinois Press, 1949), first edn.

[7] Shannon, C. E. Prediction and Entropy of Printed English. *The Bell System Technical Journal* **30**, 50–64 (1951).

[8] Kontoyiannis, I., Algoet, P. H., Suhov, Y. & Wyner, A. J. Nonparametric entropy estimation for stationary processes and random fields, with applications to English text. *Information Theory, IEEE Transactions on* **44**, 1319–1327 (1998).

[9] Manning, C. D. & Schütze, H. *Foundations of Statistical Natural Language Processing* (MIT Press, Cambridge, MA, USA, 1999).

[10] Chen, S. F. & Goodman, J. An Empirical Study of Smoothing Techniques for Language Modeling. In *Proceedings of the 34th Annual Meeting on Association for Computational Linguistics*, ACL '96, 310–318 (Association for Computational Linguistics, Stroudsburg, PA, USA, 1996).

[11] Witten, I. H. & Bell, T. The zero-frequency problem: estimating the probabilities of novel events in adaptive text compression. *Information Theory, IEEE Transactions on* **37**, 1085–1094 (1991).

[12] Brown, P. F., Della Pietra, V. J., Mercer, R. L., Della Pietra, S. A. & Lai, J. C. An Estimate of an Upper Bound for the Entropy of English. *Comput. Linguist.* **18**, 31–40 (1992).

[13] Paninski, L. Estimation of Entropy and Mutual Information. *Neural Computation* **15**, 1191–1253 (2003).

[14] Schürmann, T. & Grassberger, P. Entropy estimation of symbol sequences. *Chaos* **6**, 414–427 (1996).

[15] Miller, G. A. Note on the bias of information estimates. *Information theory in psychology: Problems and methods* **2**, 95–100 (1955).

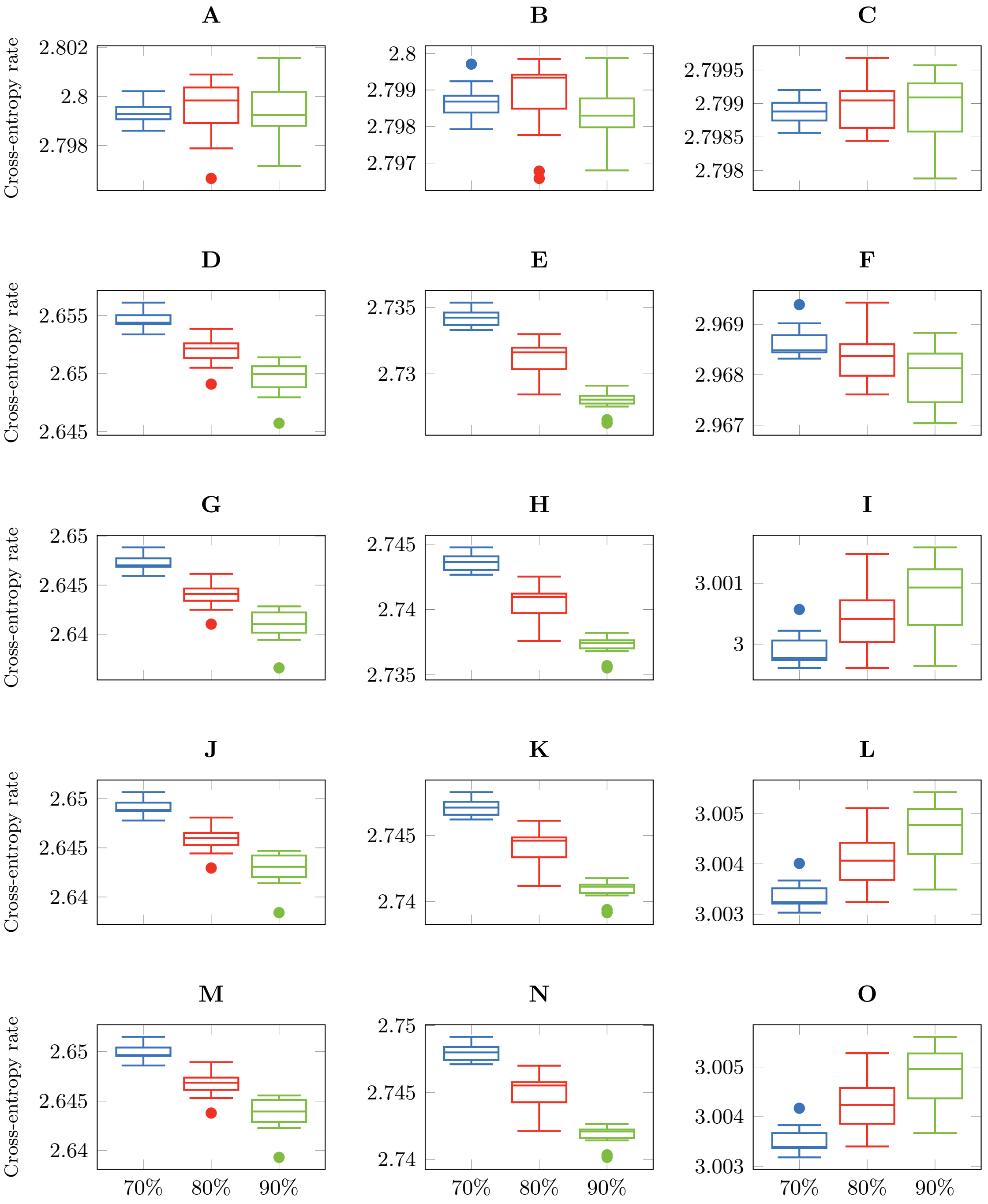

Figure 5: Convergence plots of the cross-entropy rates given the training set size (70-90%) for the fourth category level of the ICD-9 coding scheme. The rows represent the order n of the n-gram model with the first row $n = 1$ and the last row $n = 5$. The columns differentiate between the original dataset $\mathcal{D}$ in the first column, the individually shuffled disease histories $\mathcal{D}'$ (second column), and finally the population-wide shuffled dataset $\mathcal{D}''$ (third column).

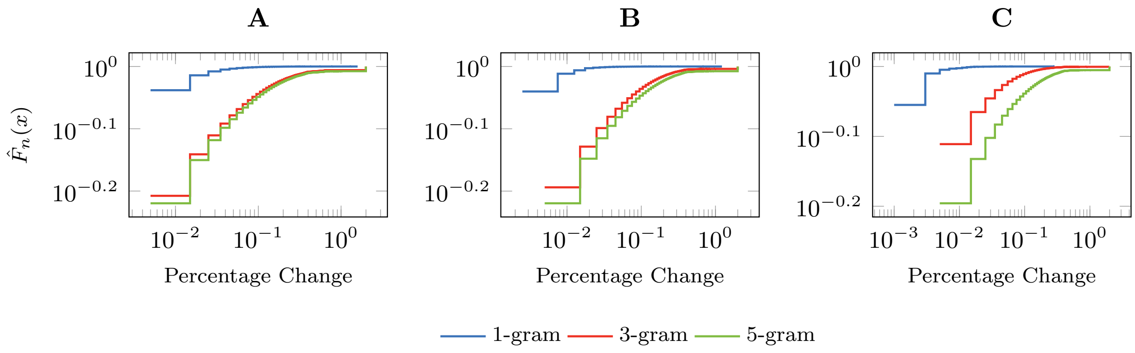

Figure 6: Cumulative distribution function of the percentage changes in log-log between the joint n-gram probabilities $q(\mathbf{b}_i)$ across the folds k and l with $k \neq l$ accounting for the frequencies at which the n-grams occur. Figure **A** gives the result for the full ICD-9 alphabet, while figure **B** and **C** represent the categories CAT_3 and CAT_2 respectively.
